# Recovery from rabies, a universally fatal disease

**DOI:** 10.1186/s40779-016-0089-y

**Published:** 2016-07-15

**Authors:** S. Manoj, A. Mukherjee, S. Johri, K. V. S. Hari Kumar

**Affiliations:** Department of Neurology, Army Hospital (R&R), New Delhi, 110011 India; Department of Neurology, Command Hospital, Lucknow, 226002 India; Department of Endocrinology, Command Hospital, Chandimandir, 134107 India

**Keywords:** Rabies, Recovery, Encephalitis, Anti-rabies serum, Mortality

## Abstract

**Background:**

Rabies is a zoonosis transmitted via the bites of various mammals, primarily dogs and bats. Known since antiquity, this disease may have the deadliest human fatality rates and is responsible for approximately 65,000 deaths worldwide per year.

**Case presentation:**

We report the case details of a 13-year-old boy from India belonging to a South Asian ethnicity, who presented with altered sensorium one month following a dog bite. He did not receive the active rabies immunization and was managed with supportive therapy. The patient had extensive T_2_W (T_2_ weighted)/fluid attenuation and inversion recovery (FLAIR) hyper intensities involving the deep gray matter of the cerebral hemispheres, hippocampus, brainstem, and cerebellum. The diagnosis was confirmed by the demonstration of the rabies antigen from a nuchal skin biopsy and a corneal smear. The patient had a slow but significant recovery over four months and was discharged from the hospital in stable condition with severe neurological sequelae.

**Conclusion:**

We report a unique case of survival after infection with a universally fatal disease.

## Background

Rabies is a zoonosis that is transmitted to humans from the bite of a rabid animal. The rabies virus is usually transmitted via dog, fox, and/or bat bites. The rabies virus is Genus *Lyssa*, a member of the *Rhabdoviridae* virus family. Rabies manifests most often as encephalitis in two clinical forms known as the furious or dumb types [1]. The former (furious) is observed in 80 % of patients, and the dumb (paralytic) variety is observed in the remaining 20 % of patients. Rabies encephalitis has the highest case fatality rates of any infectious disease and is responsible for approximately 65,000 deaths per year worldwide. India is a major contributor to the mortality rate, accounting for more than half of all deaths [2].

The medical community had renewed its interest in rabies with the publication of a case of a rabies survivor who was treated with a novel protocol known as the Milwaukee protocol (MP) [3]. Subsequent investigators who used this protocol did not find any reduction in mortality rates, and the disease still remains unconquered by modern medicine [4]. There are only 13 reported cases of rabies survivors worldwide to date; the last case was reported in India in April, 2014 [5]. We report another patient with this deadly disease who survived with the help of intensive critical care support.

## Case presentation

A 13-year-old boy from India belonging to South Asian ethnicity had sustained an unprovoked bite on his right hand from a street dog on Aug. 26, 2014. He was taken to a local hospital where the wound was cleaned, and was given the first dose of intramuscular (im) Rabipur as part of the post-exposure prophylactic (PEP) treatment. He was not given rabies immunoglobulin and received two more doses of im Rabipur on days 3 and 7 after the bite. The patient complained of headache and fever from the 10^th^ day and was treated symptomatically by the local physician. Over the next two days he started vomiting and became drowsy. He was brought to our hospital 35 days after the dog bite with an altered state of consciousness. His initial clinical examination revealed normal vital parameters with no dysautonomic features. Neurological examination showed a Glasgow Coma Scale (GCS) of 11/15 (E2 M5 V4) and intact brain stem reflexes along with signs of meningeal irritation. He had no focal neurological deficits, and a fundal examination was normal.

**Fig. 1:**
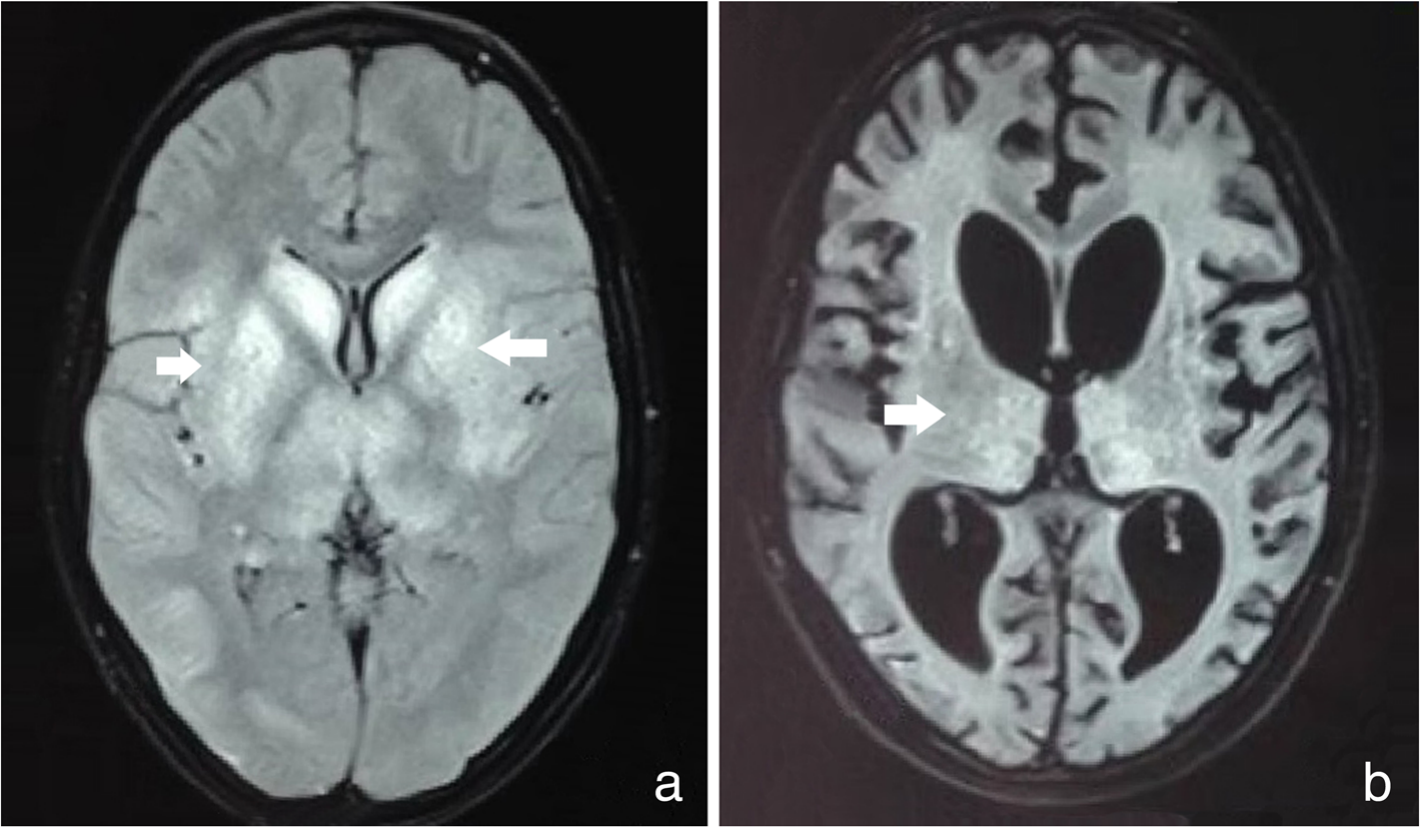
MRI images (FLAIR) performed one month after the patient was bitten; hyperintensities in the basal ganglia (*white arrows*), thalamus, pons, and medulla (**a**) and brain atrophy (**b**) are shown

He was initially treated with broad spectrum antibiotics as well as antimalarial agents in response to febrile encephalopathy in an endemic region and was investigated for possible etiologies. His hematological and biochemical parameters were normal. Screening for malaria and toxic substances, including lead and benzodiazepines, showed negative results. Cerebrospinal fluid (CSF) analysis showed lymphocytic pleocytosis (WBC-20), elevated proteins (76 mg/dl), normal glucose (60 mg/dl, blood glucose of 112 mg/dl), and negative staining (Gram stain, Acid fast bacilli, India ink preparation) of the CSF culture. The initial magnetic resonance imaging (MRI) showed bilateral thalamus and brainstem hyperintensities in the T_2_W and FLAIR images without diffusion restriction or hemorrhages on the gradient; these findings were suggestive of encephalitis (Fig. 1a).

The possible diagnoses after neuroimaging were Japanese B-, West Nile-, or rabies-associated encephalitis. The CSF screen was negative for Herpes, Japanese B, and West Nile viruses by RT-PCR. In view of the possibility of rabies encephalitis, he was evaluated using paired serum and CSF samples for antibody titers. The paired sera showed the antibody titers in excess of 1:60,000 dilution after 45 days of vaccine treatment confirming the diagnosis of rabies infection. Subsequent to these results, other samples were collected from the nape of the neck and the cornea. The presence of the rabies antigen in the nerve twigs confirmed the diagnosis of rabies encephalitis. The confirmatory tests were conducted at the Department of Virology, Armed Forces Medical College (AFMC), Pune and the National Institute for Mental Health and Neurological Sciences (NIMHANS), Bengaluru.

He was managed in the intensive care unit (ICU) with ventilator support, barrier nursing, and strict universal precautions. The ICU course was complicated by ventilator associated pneumonia, autonomic storms, and recurrent seizures. The sympathetic storm management included the use of intravenous labetalol for tachycardia and hypertension. His management included broad spectrum antibiotics for *Pseudomonas* pneumonia, prophylaxis for deep vein thrombosis, a tracheostomy, antiepileptic drugs, and aggressive physiotherapy. For approximately three weeks, he remained comatose with a motor score of 3 and a GCS score of 5 with pronounced bulbar palsy. Nutritive requirements were met by the nasogastric feeds. He was weaned off of the ventilator after eight weeks of illness.

Over the next two months he developed spontaneous eye opening and was able to follow verbal commands. Motor functions partially improved with spontaneous movements of the limbs and truncal muscles. He continued with aggressive physiotherapy, and after five months of hospitalization, he was discharged in stable condition with neurological sequelae. MRI scans were conducted at serial intervals and showed the reduction of hyperintensities along with marked cortical atrophy (Fig. 1b). During the last review (two months after discharge), the patient was able to make meaningful eye contact and follow single step commands.

## Discussion

Rabies encephalitis is almost always a fatal disease, and therapy has mostly been palliative. Prior to 2004, there were only five documented human survivors, all of whom had received the PEP, albeit incomplete or late [5]. In 2004, the first survivor without PEP was reported after this individual had undergone the MP. However, the use of the MP did not increase survival in subsequent cases [6]. Our patient was managed with aggressive supportive care without the MP or steroids. Our patient survived the acute phase of rabies encephalitis and was discharged after five months of intensive nursing care in the hospital.

The reasons for patient survival after rabies infection are always conjectural depending on the number of survivors of this disease [7]. The presence of high antibody titers in the CSF could be a strong predictor for limited neurological damage, which may help lead to survival. Unfortunately, similar data from other survivors could not be assessed; therefore, we lacked any comparative data. Another possible reason could be the intensive nursing care and proper management of the autonomic storms. Genetic variability in the host immune response to the rabies virus could also be another contributory factor to survival.

The ongoing viral effects with associated devastating brain injury were observed in serial MRI scans [8]. However, intensive supportive care allows the immune response to clear the virus while retaining the potential for reversing the neurologic consequences. This resulted in a slow recovery despite the massive initial neurological damage. Out of the seven reported rabies survivors until 2008, only the index case that used the MP did not receive PEP. Rabies virus was detected in only one case in all of the others that were diagnosed using only the rabies antibody [9]. Our case was diagnosed using both the rabies antibody and highly specific rabies antigen detection tests.

## Conclusions

In conclusion, we report the case details of a young boy who survived rabies, a disease with few survivors. Our report along with other published reports, should give more impetus to researchers to unravel the mechanisms to conquer the rabies infection.

## Abbreviations

CSF, Cerebrospinal fluid; FLAIR, Fluid attenuation and inversion recovery; GCS, Glasgow coma scale; ICU, Intensive care unit; MP, Milwaukee protocol; MRI, Magnetic resonance imaging; PEP, Post exposure prophylaxis; T_2_W: T_2_ weighted
